# CD3Ɛ immune restorative ability induced by Maitake Pro4x in immunosupressed BALBc mice

**DOI:** 10.1186/s13104-022-06201-1

**Published:** 2022-09-23

**Authors:** Diego Maximo Aguilera-Braico, Gabriela A. Balogh

**Affiliations:** 1 BIOMED-UCA, Laboratory of Biomedical Sciences, Pontifical Catholic Argentine University-UCA, 1600 Alicia Moreau de Justo Avenue, 1007 Buenos Aires, Argentina; 2 64 Jewel Lane, Levittown, Pennsylvania 19055 USA

**Keywords:** Maitake D-Fraction Pro4X, TL /NK cells, BL/stem cells, Granulocytes, Immunosuppression

## Abstract

**Objectives:**

The aim of this research was to determine if the rich beta glucan compound called Maitake Pro4X can recover the T cell/NK population depleted by Dexamethasone treatment in lymph nodes from cancer-free BALBc female mice. A CD3Ɛ molecular FITC labelled marker was used to measure the effect of Maitake D-Fraction Pro4X (5 mg/kg) on T cell/NK cells populations employing flow cytometry from immunosuppressed female BALBc mice in lymph nodes. There were employed other molecular markers such as CD19, CD105, Ly6G.

**Results:**

Maitake Pro4X (5 mg/kg) was able to recover 42.97% of the depleted CD3Ɛ FITC cell population level in Lymph nodes from immunosuppressed female BALBc mice from 4.328 ± 6.229 to 22.646 ± 12.393 (p < 0.01) using Flow Cytometry. Maitake was also able to significantly increase the Ly6G PE cell population with p < 0.05 in lymph nodes.

**Supplementary Information:**

The online version contains supplementary material available at 10.1186/s13104-022-06201-1.

## Introduction

There are many species of mushrooms have been reported as potentially useful in human health. However, has been little explored the role of purified extracts of *Grifola frondosa* edible mushroom (Maitake) on its antitumor and immune-activating potential [1–3].

In 2008, study employing mice lung-metastasis model shows that MD-Fraction was able to enhance the APC’s IL-12 production, activating NK cells, and increasing cytotoxicity against colon-carcinoma cells [4].

In 2009, a polysaccharide extract from Maitake mushroom were employed in a phase I/II clinical trial involving 34 postmenopausal breast cancer patients, showing immunomodulatory effects after 3 weeks of treatment [5]. In another study in 2015, was demonstrated that Maitake extract’s treatment (3 mg/kg) increased the endogenous (basal) neutrophil and monocyte function among other beneficial effects in 23 Myelodysplastic syndromes (MDS) patients [6]. Suggesting that Maitake extract has beneficial immunomodulatory potential in those patients. In 2016, our laboratory found that the purified extract from *Grifola frondosa* mushroom, Maitake D-Fraction Pro4X (at 5 mg/kg/day) administrated daily for 15 days was able to prevent more than 60% breast tumor development, blocked tumor invasiveness, reduced angiogenesis and increased overall survival in female BALBc mice [7]. Moreover, Alonso et al. [8], demonstrated that D-Fraction decreases tumor burden and reduces the number of lung metastases in a murine model of breast cancer, among other benefits [8]. As suggested by recent studies, MD-Fraction also exerts antiproliferative and anti-invasive potential on colon cancer cells [9].

More recently, has been reviewed that Maitake D-Fraction reduces the size of mammary, hepatic, and pulmonary cancers, while, at the same time, the purified extract Maitake D-Fraction Pro4X, exerts a significant role in reducing angiogenesis, carcinogenesis, and invasiveness [10].

To contribute more with the knowledge of Maitake D-Fraction we demonstrate in this in vivo study its role as immunomodulatory agent exerting a potentiation of the immune T cells depleted population in BALBc mice.

## Main text

The most important discovery found here was that oral treatment with 5 mg/kg/day Maitake D Fraction (Pro4X) can recover 42.97% the level of CD3Ɛ cells in Lymph nodes from Dexamethasone-treated mice. Maitake was not able to recover the depleted level of CD19 cells nor the CD105 cells in lymph nodes or spleen either. Maitake Pro4X exert an important effect on Ly6G cells, increasing its level to 771.1% and 50.2% above control in lymph nodes and Spleen tissue, respectively.

## Methods

An experimental study was design employing 21 female albino mice of the BALB/c strain (15–20 days old/20–25 g). The animals were reproduced inbred and acquired from the Animal Facility of the Biomedical Research Institute (BIOMED) from UCA-CONICET maintain in mice boxes with food and water ad libitum. Healthy animals with normal immune status were employed in this experiment, diving into 3 groups: Healthy control Group (5 mice), Dexamethasone treated Group (8 mice) and Dexamethasone (Glucocorticoid-Decadron 0.5 mg) in combination with Maitake Pro4X Group (8 mice). The animals were treated daily by oral administration of water/PBS Solution (Control Group) or 5 mg/kg of Maitake Pro4X (Maitake Group) in absence or presence of 0.15 mg/kg Dexamethasone for 3 weeks (Dexa + Maitake Group). After treatment, animals were sacrificed by CO_2_ asphyxiation as euthanasia method. Lymph nodes (LNs) and spleen tissue (ST) were removed. LNs were collected from inguinal, mesenteric, and axillary mice lymph nodes. White blood cells were isolated employing an Fisherbrand Sterile Cell strainer to obtain 8–9 × 10^6^ cells suspension/tube. Cells were isolated and washed with PBS (phosphate saline buffer) and resuspended at 400–500 cells/condition with a Sorting Buffer (1 × Phosphate Buffered Saline (Ca/Mg^2+^ free), 1 mM EDTA, 25 mM HEPES pH 7.0, 1% Fetal Bovine Serum (Heat-Inactivated) and labelled with the following fluorescent-labelled antibodies: Anti-CD3Ɛ Antibody (145-2C11) FITC-conjugated Antibody (Santa Cruz Biotechnology, Texas, USA), anti-mouse CD19 PE-conjugated (Clone No. PeCa1) (Immunotools Inc, Germany), CD105 Alexa Fluor Conjugated Antibody (Clone No. MJ7/18) (Bio Rad, California, USA) and Ly6G FITC (Fluorescein) Conjugated Antibody (Clone No. 1A8) (ABCAM, Cambridge, UK) for the study of LT/NK cells, LB/Dendritic cells/Stem cells, Macrophages/ Monocytes/Stem cells or Granulocytes, respectively.

For cell sorting in immunological studies a FACS (fluorescence activated cell sorting) technique was employed to applied in BD Accuri C6 Flow Cytometer Equipment.

To perform the Cell Surface Target Antibody Staining for Flow Cytometry, 500 µL of cells suspended were incubated with 150 µL each primary antibody (described before) diluted 1:30 with PBS + BSA 0.5% Buffer. Incubated 1 h in dark, and performed 3 washes with PBS + BSA 0.5%, centrifuged samples, resuspend cell pellet using 400 µL PBS + BSA 0.5% and proceed immediately to flow cytometer BD Accuri™ C6. Sample duplicate were run. The data was acquired on logarithmic scale and were analyzed by FlowJo Software (Tree Star, OR, USA).

## Results

**Fig. 1:**
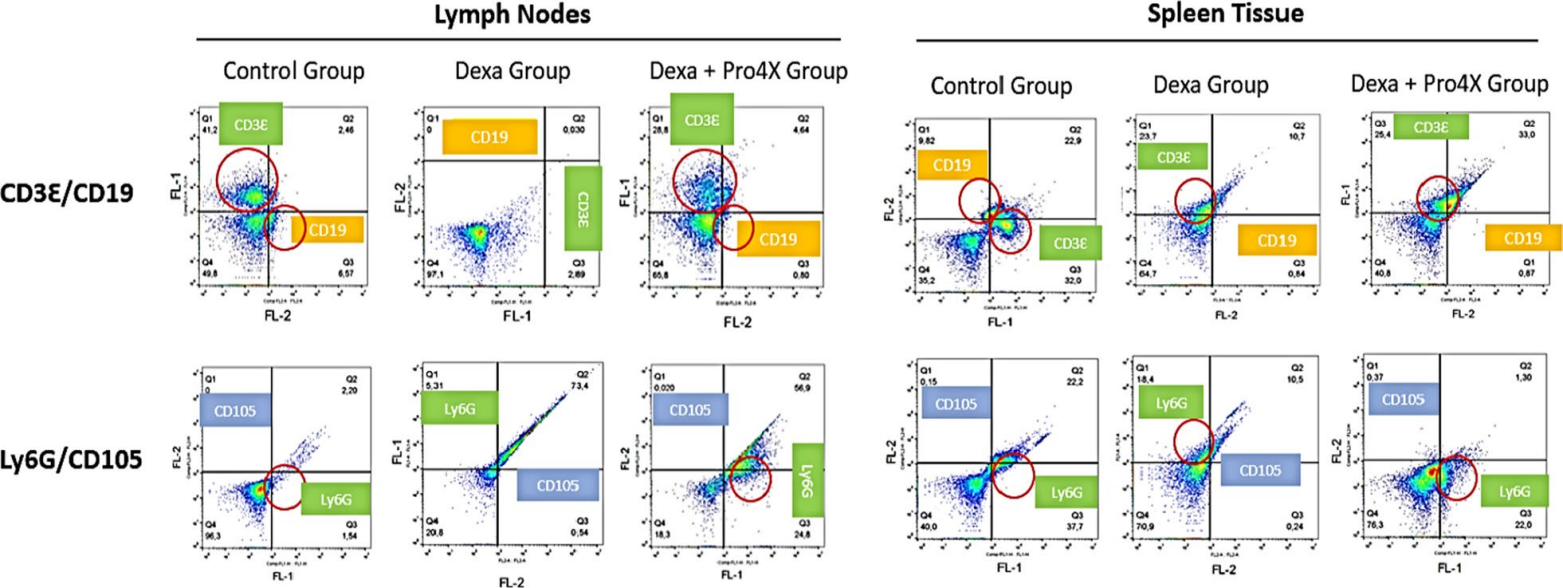
The flow cytograms for the molecular markers CD3Ɛ, CD19, Ly6G and CD105 from lymph nodes and spleen tissue isolated from BALBc mice employed in this study classified into three groups: Control Group, Immunosuppressed Group (Dexa Group) and Immunosuppressed + Maitake Pro4X Group (Dexa + Pro4X Group)

A representative Flow Cytograms from each molecular marker and each condition in LNs and Spleen are shown in Fig. 1. Data was analyzed using the FlowJo Software and T-Student test was applied for the statistical calculations.

**Fig. 2:**
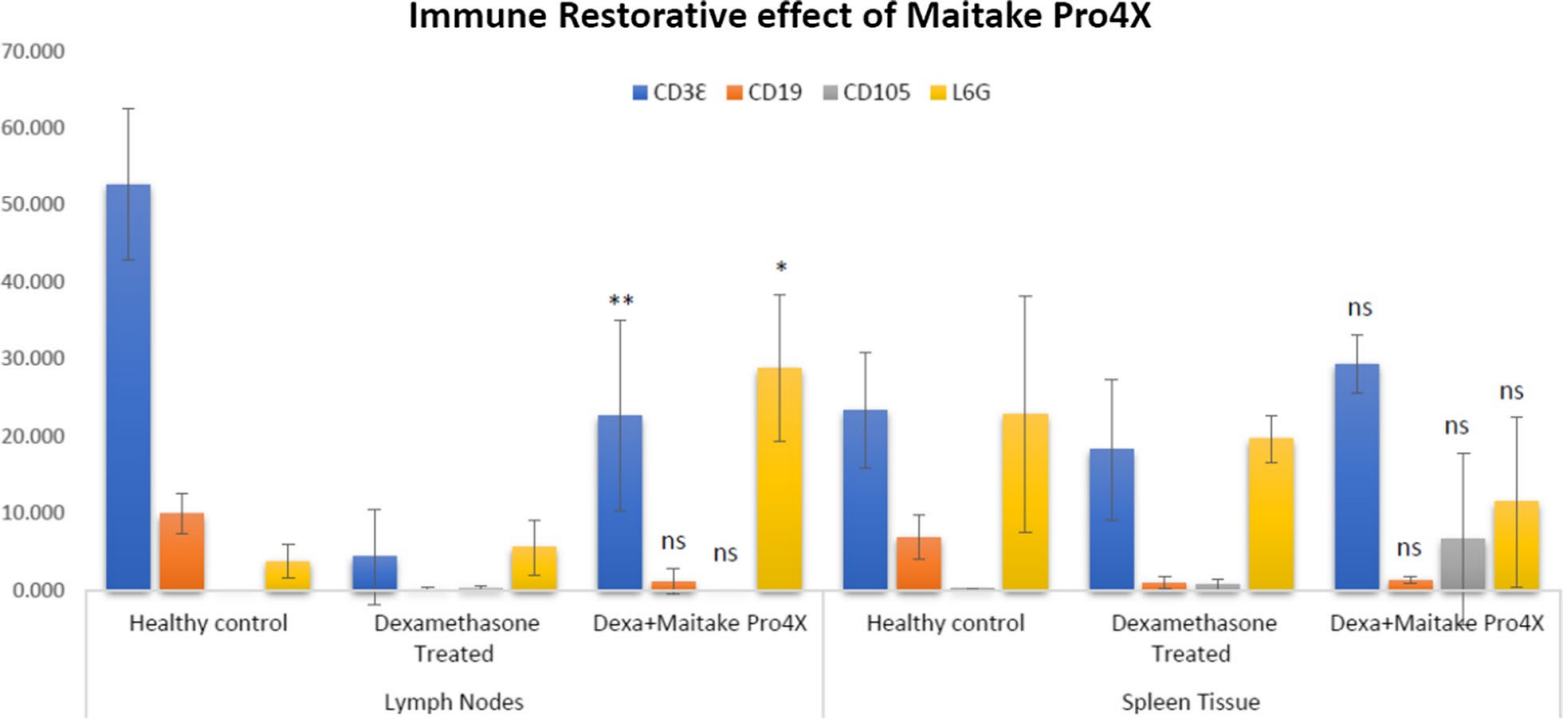
Flow cytometry quantification of immune cells in Lymph nodes and spleen tissue from immunosuppressed BALBc mice treated with or without Maitake Pro4X. The graphics represent the mean and its SD of the analyzed flow cytometry data using the FlowJo Software. In the graphic * represent p < 0.05 and ** represent p < 0.005 respect to the Dexamethasone treated condition. ns means statistically not significance were found in that condition with respect to immunosuppressed, dexamethasone treated group

**Fig. 3:**
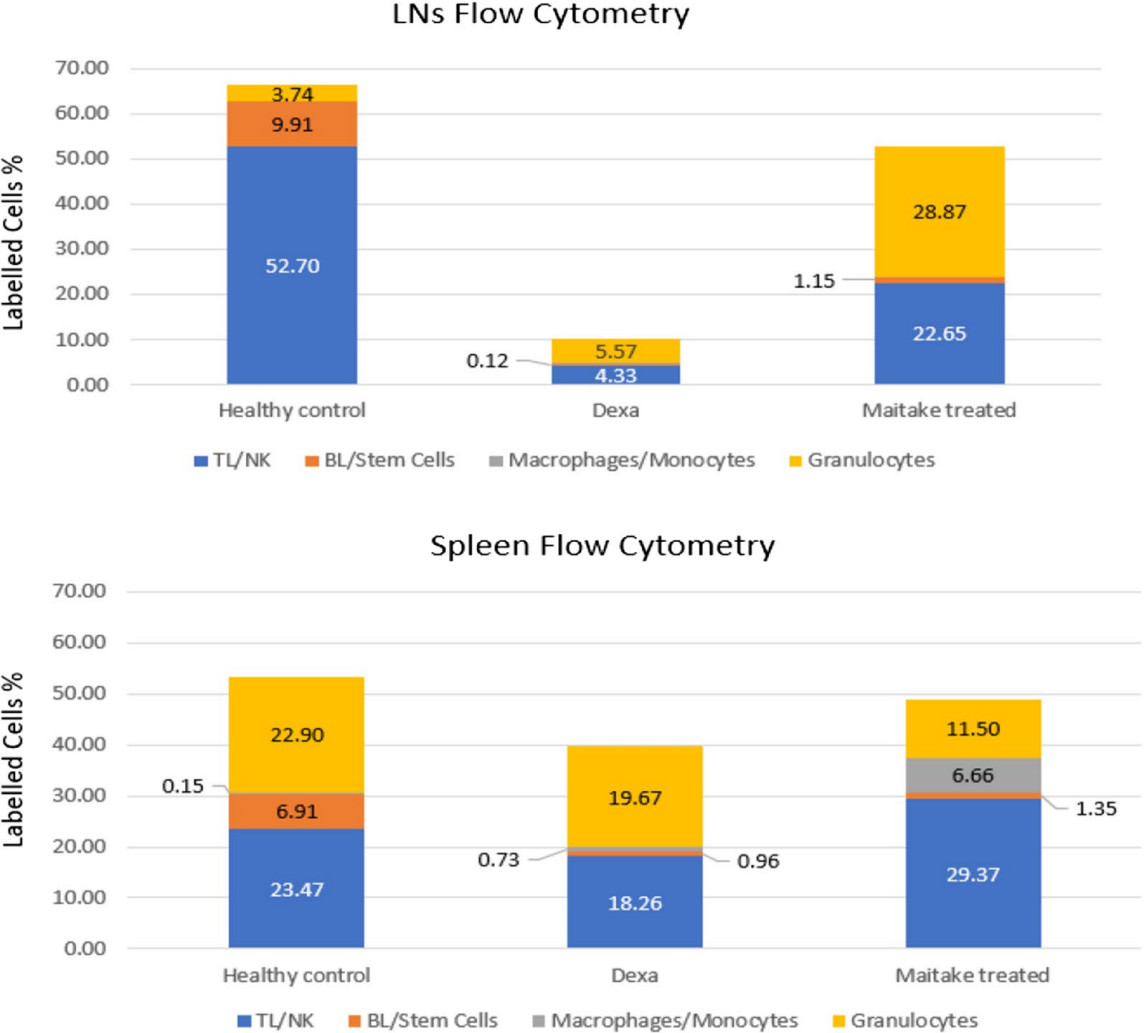
Percentage of immune cells present at healthy control, and during dexamethasone treatment with and without Maitake D-Fraction Pro4X at Lymph nodes and spleen tissue from BALBc mice. In the graphic, blue columns represent TL/NK cells, orange columns represent BL/stem cells, gray columns represent Macrophages/Monocytes/ and dark yellow columns representing the % of granulocytes labelled cells detected on the flow cytometry analysis

The flow cytometry quantification of immune molecular markers in each condition from LNs and spleen tissue in BALBc mice is shown in Fig. 2. The results shown that the healthy control BALBc mice have a different and variable percentage of immune cells in lymph nodes compare to spleen tissue. In LNs were observed 79% of TL/NK Cells, 14.9% BL/stem Cells, 0.0% of macrophages/monocytes and 5.6% of granulocytes cells. However, in spleen were observed 43.92% TL/NK cells, 12.93% BL/stem cells, 0.28% macrophages–monocytes and 42.86% granulocytes (Fig. 3).

There were also observed in graphics from Fig. 3 in LNs that the immunosuppressor dexamethasone deplete 91.78% (from 52.70 ± 9.85 to 4.33 ± 6.23, p < 0.005) the level of TL/NK cells, and 98.79% (from 9.91 ± 2.62 to 0.12 ± 0.22, p < 0.005) (Additional file 1: Table S1) the BL/Stem cells (Additional file 2: Table S2) but did not affect the level of Macrophages/Monocytes (p > 0.05) (Additional file 3: Table S3) or Granulocytes cells (p > 0.05) (Additional file 4: Table S4) in mice lymph nodes. By another hand, were observed that dexamethasone treatment are seem to be less effective at spleen tissue level, reduce only 22.19% (from 23.47 ± 7.49 to 18.26 ± 9.17) TL/NK cells level (Additional file 1: Table S1), decreased in 86.11% the level of BL/stem cells (from 6.91 ± 2.88 to 0.96 ± 0.77) (Additional file 2: Table S2), reduce only 14.10% de level of Granulocytes (from 22.90 ± 15.32 to 19.67 ± 3.00) (Additional file 4: Table S4) and did not change the level of Macrophages/Monocytes (Additional file 3: Table S3), however none of this changes were statistically significant (p > 0.05) (Fig. 3). In the concomitant treatment of Maitake Pro4X and dexamethasone in lymph nodes were observed that Pro4X was able to recover about 42.97% the level of TL/NK cells with respect to the healthy control from 52.70 ± 9.85 to 22.65 ± 12.39 with p < 0.005 (Fig. 3) (Additional file 1: Table S1). Maitake treatment did not recover BL/Stem Cells level with respect to healthy control from 9.91 ± 2.62 to 1.15 ± 1.68 p > 0.05 (Additional file 2 : Table S2). There were not observed any significant changed in the level of Macrophages/Monocytes between the normal control and Maitake treatment (from 0.00 ± 0.00 to 0.012 ± 0.010) (p > 0.05) (Additional file 3: Table S3). Maitake Pro4X recover more than 700% the level of Granulocytes cells with respect to healthy control from 3.74 ± 2.20 to 28.87 ± 9.57 with p < 0.05) (Fig. 3) (Additional file 4: Table S4).

In the spleen tissue were observed that Maitake Pro4X did not significantly changed the level of TL/NK cells with respect healthy control from 23.47 ± 7.49 to 29.37 ± 3.77, p > 0.05 (Additional file :1 Table S1). Maitake did not recover the depleted BL/stem cells level with respect to controls (6.91 ± 2.88 to 1.35 ± 0.43, p > 0.05) (Additional file 2: Table S2). Pro4X also failed to recover the depleted granulocytes level from 22.90 ± 15.32 to 11.50 ± 11.03, p > 0.05) (Additional file 4: Table S4). However, were observed a spectacular recovery in more than 4000 times with respect to controls in the depleted macrophages–monocytes level from 0.15 ± 0.15 to 6.66 ± 11.21 but that difference was not statistically significant (p > 0.05) (Additional file 3: Table S3) (Fig. 3).

## Conclusions and discussion

In this study were observed different levels of immune cells in lymph nodes and spleen tissue in normal/healthy BALBc mice group. In lymph nodes (LNs) there are almost 80% TL/NK cells and in Spleen there are about 40% TL/NK cells and similar % in granulocytes.

Dexamethasone can alter the normal responses of the immune system and, therefore, be useful in the treatment of certain diseases that affect the immune system, such as anemia (aplastic and hemolytic), thrombocytopenia and purple. Corticosteroids are used to treat rheumatoid arthritis, inflammatory bowel disease, and many other conditions. These drugs also help suppress the immune system to prevent organ rejection in transplant recipients. GCs (glucocorticoids) are fat-soluble molecules, are easily absorbed on any skin or mucosal surface, circulate in the blood, bound to proteins and the free fraction is the one that diffuses inside the cells, exerting its action. Although the simple diffusion mechanism through the lipid bilayer of cell membrane is the most accepted, there is evidence that its entry into the cell is regulated through membrane receptors other than the steroid receptor (GR). They would use G proteins as signals and this mechanism would be responsible for the rapid actions of these hormones [11]. However, the exact molecular mechanism of GCs such as dexamethasone are not yet fully elucidated.

In this study were observed that dexamethasone treatment did not deplete the level of immune cells in Spleen but affected more than 92% the TL/NK and BL cells level in Lymph nodes. As was reported in 2018, Dexamethasone-mediated T cell suppression diminishes naïve T cell proliferation and differentiation [12], these findings are in accordance with our results.

Were also observed that the treatment with dexamethasone did not deplete the level of dranulocytes (neutrophils, basophils and eosinophils) nor either in LNs or spleen tissue. These results are partially agreed with a discovery performed in 1999, explained by the fact that glucocorticoids are known to negatively modulate apoptosis of human neutrophils [13]; however, it is still unclear by which mechanism delay the granulocytes death.

Beta-glucans are naturally occurring polysaccharides, glucose polymers are constituents of the cell wall of certain pathogenic bacteria and fungi. These mushrooms contain biologically active polysaccharides that mostly belong to beta-glucans groups, that increase host immune defense by activating complement system, enhancing macrophages and natural killer cell functions [14]. Maitake (*Grifola frondosa*) has been prized in traditional Japanese herbology for hundreds of years. Modern science has identified in Maitake Proteoglycan, beta-1,3 and 1,6 glucans, as an active constituent to support the immune system. Maitake D-fraction is a standardized form of pure and active proteoglycan, contains 6000 mg of extract from Maitake mushroom (PD-Fraction TM) in alcohol free vegetable glycerin base.

In our studies were observed that the Maitake Pro4X treatment acts antagonistically with the immunosuppressor dexamethasone, recovering more than 40% the level of depleted TL/NK cells but are unable to recover the depleted level of BL/stem cells, however, surprisingly increased more than 700 times the granulocytes level in lymph nodes. Meaning that, under immune-depletion condition, Maitake Pro4X activate the production of granulocytes cells in lymph nodes ready to blood circulation to protect the host, also were observed in this study that Maitake Pro4X significantly help recovering in about 40% the completed depleted level of TL/NK cells in lymph nodes due to dexamethasone treatment. A study performed in 2010, indicates that oral MBG (Maitake mushroom *Grifola frondosa*) promoted maturation of HPC (human hematopoietic progenitor cells) to become functionally active myeloid cells and enhanced peripheral blood leukocyte recovery after chemotoxic bone marrow injury [15].

The conclusion in the present study opens a light to see good potential for beta-glucans from Maitake Pro4X in the immune system of mice, and it still interesting to considered Maitake Pro4X as immunosuppressor antagonist agent in patients with certain clinical conditions, contrasting the negative effect of such treatments, and recovering the level of the white cells for the host immune defense. Regarding the immune system, the regulation of its activation or suppression could contribute to the maintenance of a good state of host health. The use of agents that activate host defense mechanisms (immunostimulatory, immunopotentiators or biological response modifiers) could provide an additional therapeutic tool to conventional chemotherapy.

Based on these results, now will be interesting to understand by which molecular mechanism the beta-glucans from Maitake Pro4X are able to recover the Dexamethasone-depleted immune cells.

## Limitations

Findings from our research were performed in small number of mice. In addition, we uses 4 labelled molecular markers to analyze the most common immune cells.

## Supplementary Information

**Additional file 1: Table S1.** CD3Ɛ FITC labelled cell population in lymph node and spleen from BALBc mice

**Additional file 2: Table S2.** CD19 PE labelled cell population in lymph node and spleen from BALBc mice

**Additional file 3: Table S3.** CD105 AF labelled cell population in lymph node and spleen from BALBc mice

**Additional file 4: Table S4.** L6G FITC labelled cell population in lymph node and spleen from BALBc mice.

## Data Availability

All data generated or analyzed during this study are included in this published article (Additional file 1: Table S1, Additional file 2: Table S2, Additional file 3: Table S3 and Additional file 4: Table S4). By exercising the Licensed Rights, we (GAB and DMAB) accept and agree to be bound by the terms and conditions of this Creative Commons Attribution 4.0 International Public License.

## Abbreviations

PBS: Phosphate saline buffer
CD3Ɛ: Is a 20 kD transmembrane protein, also known as CD3 or T3. It is a member of the Ig superfamily and primarily expressed on T cells, NK-T cells, and at different levels on thymocytes during T cell differentiation
CD19: CD19 Antibody (B-1) is a high quality monoclonal CD19 antibody (also designated B-lymphocyte antigen CD19 antibody) suitable for the detection of the CD19 protein of mouse, rat and human origin
CD105: CD105 or Endoglin is a Type I transmembrane protein, which is highly expressed on human vascular endothelial cells
Ly6G: The monoclonal antibody 1A8-Ly6g reacts with mouse Ly-6G, which is also known as Gr-1. Ly-6G is a 25-kDa GPI protein expressed exclusively by neutrophils. Studies suggest an important role in neutrophil infiltration, recruitment, and migration
LNs: Lymph nodes
TL: T lymphocyte
NK: Natural killer cells
BL: B lymphocyte

## References

[1] Kodama N, Murata Y, Asakawa A, Inui A, Hayashi M, Sakai N, Nanba H (2005). Maitake D-Fraction enhances antitumor effects and reduces immunosuppression by mitomycin-C in tumor-bearing mice. Nutrition.

[2] Mayell M (2001). Maitake extracts and their therapeutic potential. Altern Med Rev.

[3] He X, Wang X, Fang J, Chang Y, Ning N, Guo H, Huang L, Huang X, Zhao Z (2017). Polysaccharides in *Grifola frondosa* mushroom and their health promoting properties: a review. Int J Biol Macromol.

[4] Masuda Y, Murata Y, Hayashi M (2008). Inhibitory effect of MD-fraction on tumor metastasis: involvement of NK cell activation and suppression of intercellular adhesion molecule (ICAM)-1 expression in lung vascular endothelial cells. Biol Pharm Bull.

[5] Deng G, Lin H, Seidman A (2009). A phase I/II trial of a polysaccharide extract from *Grifola frondosa* (Maitake mushroom) in breast cancer patients: immunological effects. J Cancer Res Clin Oncol.

[6] Wesa KM, Cunningham-Rundles S, Klimek VM (2015). Maitake mushroom extract in myelodysplastic syndromes (MDS): a phase II study. Cancer Immunol Immunother.

[7] Roldan-Deamicis A, Alonso E, Brie B, Aguilera Braico D, Balogh GA (2016). Maitake Pro4X has anti-cancer activity and prevents oncogenesis in BALBc mice. Cancer Med.

[8] Alonso EN, Ferronato J, Gandini NA, Fermento ME, Obiol DJ, Lopez Romero A, Arevalo J, Villegas E, Facchinetti MM, Curino AC (2017). Antitumoral effects of D-fraction from *Grifola Frondosa* (Maitake) Mushroom in breast cancer. Nutr Cancer.

[9] Roca-Lema D, Martinez-Iglesias O, de Ana F, Portela C, Rodríguez-Blanco A, Valladares-Ayerbes M, Díaz-Díaz A, Casas-Pais A, Prego C, Figueroa A (2019). In Vitro anti-proliferative and anti-invasive effect of polysaccharide-rich extracts from *Trametes versicolor* and *Grifola frondosa* in colon cancer cells. Int J Med Sci.

[10] Panda SK, Sahoo G, Swain SS, Luyten W (2022). Anticancer activities of mushrooms: a neglected source for drug discovery. Pharmaceuticals.

[11] Iwasaki Y, Aoki Y, Katahira M, Oiso Y, Saito H (1997). Nongenomic mechanisms of glucocorticoid inhibition of adrenocorticotropin secretion: possible involvement of GTP-binding protein. Biochem Biophys Res Commun.

[12] Giles AJ, Hutchinson MKND, Sonnemann HM, Jung J, Fecci PE, Ratnam NM, Zhang W, Song H, Bailey R, Davis D, Reid CM, Park DM, Gilbert MR (2018). Dexamethasone-induced immunosuppression: mechanisms and implications for immunotherapy. J Immunother Cancer.

[13] Lina R, James S, Gabriel B, Diana G, Patiño PJ (1999). Efecto de la dexametasona sobre la apoptosis en polimorfonucleares neutrófilos humanos inducida por especies reactivas del oxígeno. Rev Asoc Colomb Alerg Inmunol.

[14] Akramiene D, Kondrotas A, Didziapetriene J, Kevelaitis E (2007). Effects of beta–glucans on the immune system. Medicina (Kaunas).

[15] Lin H, de Stanchina E, Zhou XK, Hong F, Seidman A, Fornier M, Wei-Lie X, Kennelly EJ, Wesa K, Cassileth BR, Cunningham-Rundles S (2010). Maitake beta–glucan promotes recovery of leukocytes and myeloid cell function in peripheral blood from paclitaxel hematotoxicity. Cancer Immunol Immunother.

