# Supplementary material for: CD3Ɛ immune restorative ability induced by Maitake Pro4x in immunosupressed BALBc mice
Source: BMC Res Notes. 2022 Sep 23;15:307. doi: 10.1186/s13104-022-06201-1 (PMC9502923; doi:10.1186/s13104-022-06201-1)
Supplement: Supplementary file 1 — Additional file 1: Table S1. CD3Ɛ FITC labelled cell population in lymph node and spleen from BALBc mice [file 13104_2022_6201_MOESM1_ESM.pdf]

<u>Table 1</u>: CD3Ɛ FITC labelled cell population in Lymph node and Spleen from BALBc mice.

Conditions	Healthy control (HC)	Dexamethazone Treated (DT)	Dexa+Maitake Pro4X (MT)
CD3Ɛ In Lymph nodes	65.6	2.5	10.2
	45.6	17.5	20.3
	41.2	2.89	5.07
	58.9	9.97	28.8
	52.2	0.83	12.4
	NA	0.58	37.4
	NA	0.01	31.3
	NA	0.34	35.7
Mean	52.700	4.328	22.646
SD	9.846	6.229	12.393
Analysis Respect HC	NA	0.0015	0.0019
p value	NA	**p<0.005	**p<0.005
Analysis Respect DT	NA	NA	0.0089
p value	NA	NA	*p<0.01
CD3 Ɛ In Spleen	18.00	23.40	29.80
	20.40	23.70	25.40
	32.00	7.67	32.90
	NA	NA	NA
	NA	NA	NA
Mean	23.467	18.257	29.367
SD	7.487	9.170	3.769
Analysis Respect HC	NA	0.6410	0.2046
p value	NA	ns p>0.5	ns p>0.5
Analysis Respect DT	NA	NA	0.2623
p value	NA	NA	ns p>0.5

NA not applicable
* p value<0.01
** p value<0.005
ns no significative
