# Supplementary material for: CD3Ɛ immune restorative ability induced by Maitake Pro4x in immunosupressed BALBc mice
Source: BMC Res Notes. 2022 Sep 23;15:307. doi: 10.1186/s13104-022-06201-1 (PMC9502923; doi:10.1186/s13104-022-06201-1)
Supplement: Supplementary file 2 — Additional file 2: Table S2. CD19 PE labelled cell population in lymph node and spleen from BALBc mice [file 13104_2022_6201_MOESM2_ESM.pdf]

Table 2: CD19 PE labelled cell population in Lymph node and Spleen from BALBc mice.

Conditions	Healthy control (HC)	Dexamethazone Treated (DT)	Dexa+Maitake Pro4X (MT)
CD19 In Lymph nodes	13.6	0.00	1.15
	8.46	0.00	5.12
	6.57	0.00	0.34
	10.4	0.00	0.80
	10.5	0.00	1.31
	NA	0.58	0.01
	NA	0.34	0.44
	NA	0.01	0.01
Mean	9.906	0.116	1.146
SD	2.620	0.222	1.677
Analysis Respect HC	NA	0.0011	0.0063
p value	NA	**p<0.005	*p<0.01
Analysis Respect DT	NA	NA	0.1432
p value	NA	NA	ns p>0.5
CD19 In Spleen	4.07	1.78	1.49
	6.84	0.84	0.87
	9.82	0.26	1.69
	NA	NA	NA
	NA	NA	NA
Mean	6.910	0.960	1.350
SD	2.876	0.767	0.428
Analysis Respect HC	NA	0.1052	0.0750
p value	NA	ns p>0.05	ns p>0.05
Analysis Respect DT	NA	NA	0.0750
p value	NA	NA	ns p>0.05

NA not applicable
* p value<0.01
** p value<0.005
ns no significative
