# Supplementary material for: CD3Ɛ immune restorative ability induced by Maitake Pro4x in immunosupressed BALBc mice
Source: BMC Res Notes. 2022 Sep 23;15:307. doi: 10.1186/s13104-022-06201-1 (PMC9502923; doi:10.1186/s13104-022-06201-1)
Supplement: Supplementary file 3 — Additional file 3: Table S3. CD105 AF labelled cell population in lymph node and spleen from BALBc mice [file 13104_2022_6201_MOESM3_ESM.pdf]

Table 3: CD105 AF labelled cell population in Lymph node and Spleen from BALBc mice

Conditions	Healthy control (HC)	Dexamethazone Treated (DT)	Dexa+Maitake Pro4X (MT)
CD105 In Lymph nodes	0	0.02	0.015
	0	0.54	0.000
	0	0.35	0.020
	NA	NA	NA
	NA	NA	NA
	NA	NA	NA
	NA	NA	NA
	NA	NA	NA
Mean	0.000	0.303	0.012
SD	0.000	0.263	0.010
Analysis Respect HC	NA	0.1839	0.1917
p value	NA	ns p>0.05	ns p>0.05
Analysis Respect DT	NA	NA	0.2018
p value	NA	NA	p>0.05
CD105 In Spleen	0.005	0.4	0.37
	0.15	0.24	0.00
	0.3	1.56	19.60
	NA	NA	NA
	NA	NA	NA
Mean	0.152	0.733	6.657
SD	0.148	0.720	11.211
Analysis Respect HC	NA	0.2388	0.4163
p value	NA	ns p>0.05	ns p>0.05
Analysis Respect DT	NA	NA	0.4313
p value	NA	NA	ns p>0.05

NA not applicable
ns no significative
