# Supplementary material for: CD3Ɛ immune restorative ability induced by Maitake Pro4x in immunosupressed BALBc mice
Source: BMC Res Notes. 2022 Sep 23;15:307. doi: 10.1186/s13104-022-06201-1 (PMC9502923; doi:10.1186/s13104-022-06201-1)
Supplement: Supplementary file 4 — Additional file 4: Table S4. L6G FITC labelled cell population in lymph node and spleen from BALBc mice. [file 13104_2022_6201_MOESM4_ESM.pdf]

<u>Table 4</u>: L6G FITC labelled cell population in Lymph node and Spleen from BALBc mice

**Conditions**	**Healthy control (HC)**	**Dexamethazone Treated (DT)**	**Dexa+Maitake Pro4X (MT)**
L6G In Lymph nodes	5.95	2.16	39.80
	1.54	5.31	22.00
	3.74	9.24	24.80
	NA	NA	NA
	NA	NA	NA
	NA	NA	NA
	NA	NA	NA
	NA	NA	NA
Mean	3.743	5.570	28.867
SD	2.205	3.547	9.571
Analysis Respect HC	NA	0.4909	0.0114
p value	NA	ns p>0.05	* p<0.05
Analysis Respect DT	NA	NA	0.0168
p value	NA	NA	* p<0.05
L6G In Spleen	7.10	23.10	22.00
	37.70	18.40	12.50
	23.90	17.50	0.00
	NA	NA	NA
	NA	NA	NA
Mean	22.900	19.667	11.500
SD	15.324	3.007	11.034
Analysis Respect HC	NA	0.7380	0.3548
p value	NA	ns p>0.05	ns p>0.05
Analysis Respect DT	NA	NA	0.2838
p value	NA	NA	ns p>0.05

NA not applicable
ns no significative

*p<0.05
